# The face-cube illusion by Jean Beuchet

**DOI:** 10.1177/20416695241285911

**Published:** 2024-10-09

**Authors:** Frédéric Devinck, Christophe Quaireau

**Affiliations:** Department of Psychology, 27080 Université Rennes, Rennes, France

**Keywords:** 3D perception, history of vision science, Illusion, perception

## Abstract

The face-cube illusion was made by Jean Beuchet in 1966 (as indicated in the device) and this effect was not published. For this reason, it seems important to present this visual phenomenon. The effect is obtained from connected curved wire construction presented in three-dimensional space. The orientation of wires can be modified, and it can be perceived as either a cube or a face depending on one's viewing point.

Jean Beuchet (1914–2019) was assistant professor to the department of psychology at the University of Rennes (1949–1979). One famous visual phenomenon is the Beuchet chair that is regularly used by educational institutions and visitor attractions across the world. It consists of two parts, spatially separated, the seat and the frame. Looking from an observation point in monocular viewing, an observer combines both parts into a normal chair. From this viewing point, anyone on the seat appears to be much smaller than someone standing to its side (Beuchet, 1963a; Wiseman, 2016).

Jean Beuchet was interested in building experimental devices in visual perception with the help of Joseph Danjou, a laboratory technician who was very skilled in many fields. Unfortunately, he did not systematically publish his findings in scientific journals, but rather he used movies to communicate his work: as was done for the chair illusion and the square-circle effect (Beuchet, 1963a, 1963b).

In 1996, the laboratory of experimental psychology in Rennes celebrated its centenary. A symposium was organized, and Jean Beuchet presented the history of the experimental laboratory at Rennes and his work (Beuchet, 1996). During his personal communication, he mentioned a device named the face-cube illusion that he presented briefly for French television broadcasting in 1968 (the duration of the scene is approximately 30 s, starting at 28 min, and 40 s) (Klementieff, 1968). Since these presentations, the apparatus was never shown or discussed, thus, it seems important to present the face-cube illusion.

The face-cube device is shown in Figure 1. The apparatus is found inside a wooden box (36 cm × 36 cm × 29 cm in height) with a handle. The box can be opened from the top and two juxtaposed sides. The pattern is composed of multiple curved wires (19 cm × 23 cm × 21 cm in height). It is important to note that there are no straight wires and no right angles (see Figure 1b). All of them are connected in three-dimensional (3D) space and form a unique pattern.

**Figure 1.**
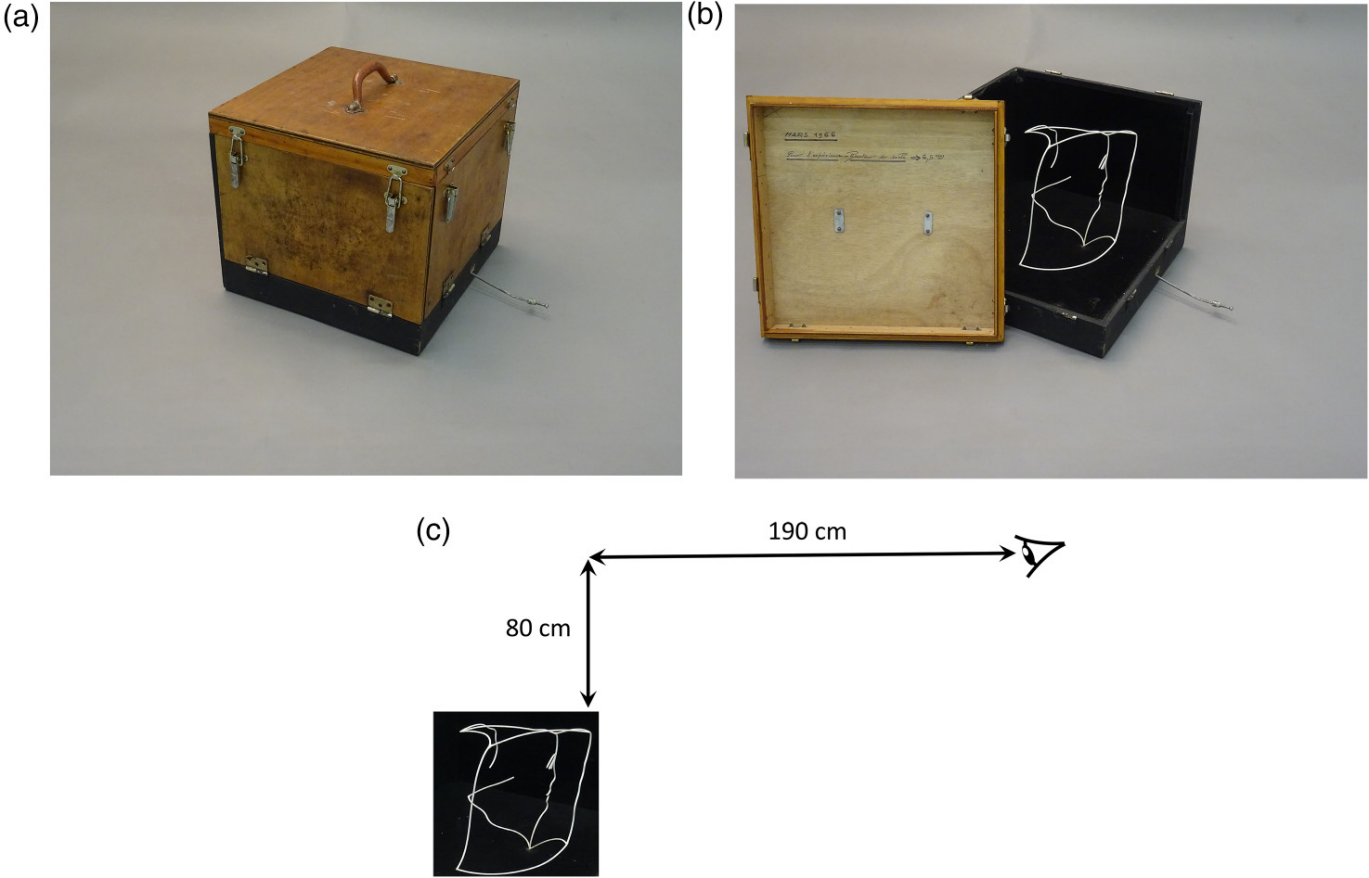
Presentation of the face-cube device. (a) The wooden box in which the face-cube is enclosed. (b) The curved wire construction. (c) Viewing distance.

Observers are positioned at approximately a distance of 190 cm from the curved wires, and the pattern is seen from above at a height of 80 cm from the top of the curved wires (Figure 1c). At the base of the box, an iron control rod allows a rotation to be applied to the pattern, which shifts the orientation of the wires to obtain different perspectives. The effect is well perceived in binocular viewing, but a monocular viewing optimizes the phenomenon.

From one viewing point, the wires in 3D space looked like a woman's face. This face in profile appeared with curved wires. When the rod at the base of the box is pushed, the pattern changes its orientation and observers have another perspective. The intermediate shape is a jumble of connected curved wires. When the rod is pushed to the end, all curved wires become straight, and a cube is perceived. An example of all points of views is presented in Figure 2 (or Movie 1). The face-cube illusion proved challenging to design, but Jean Beuchet did not indicate to us how he built the face-cube illusion.

**Figure 2.**
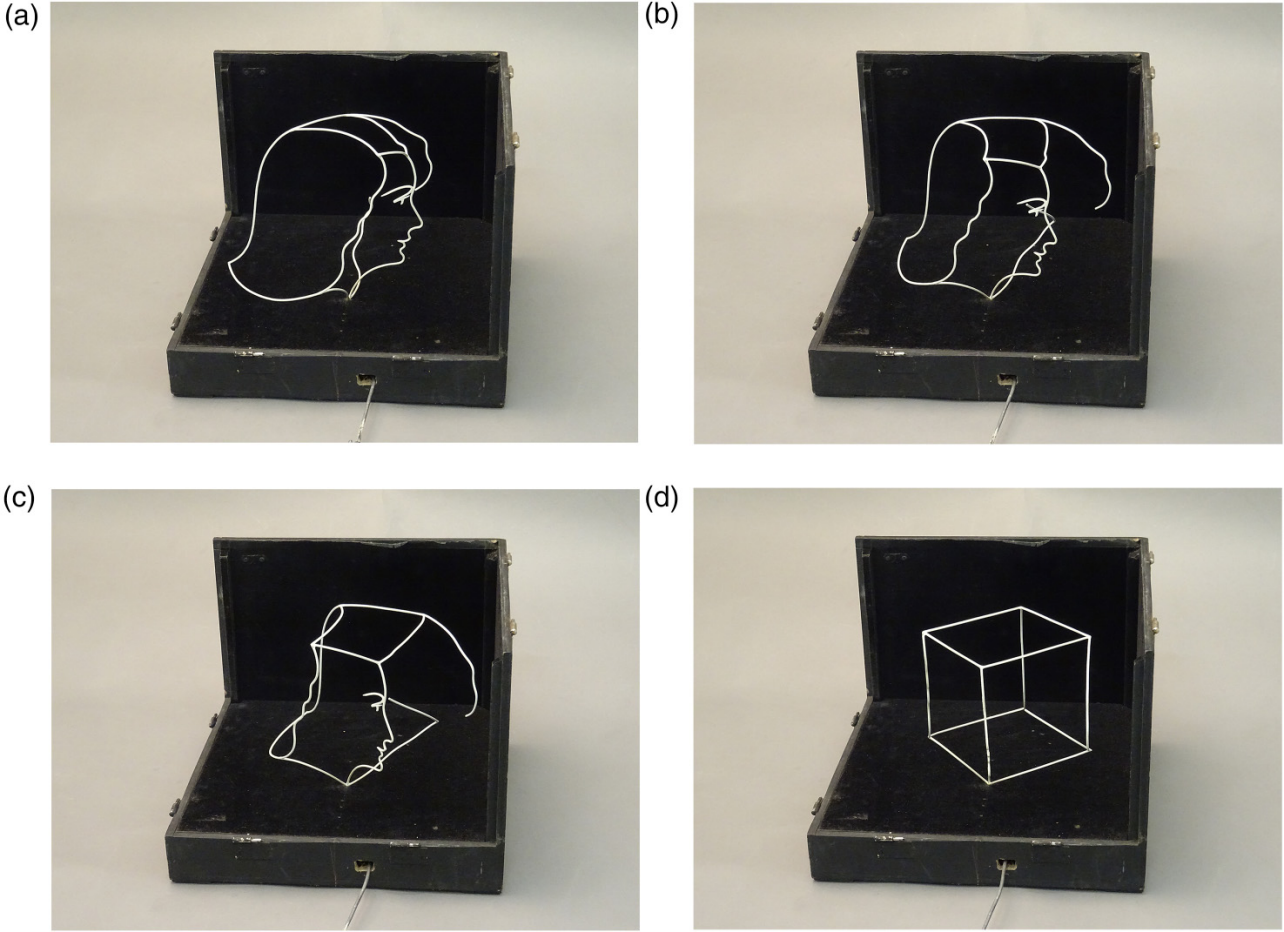
Presentation of the phenomenon in pushing the rod at the bottom of the box. (a) A woman's face in profile is perceived, (b) and (c) intermediate positions composed of curved wires, then (d) a cube is seen.

**Movie 1.**
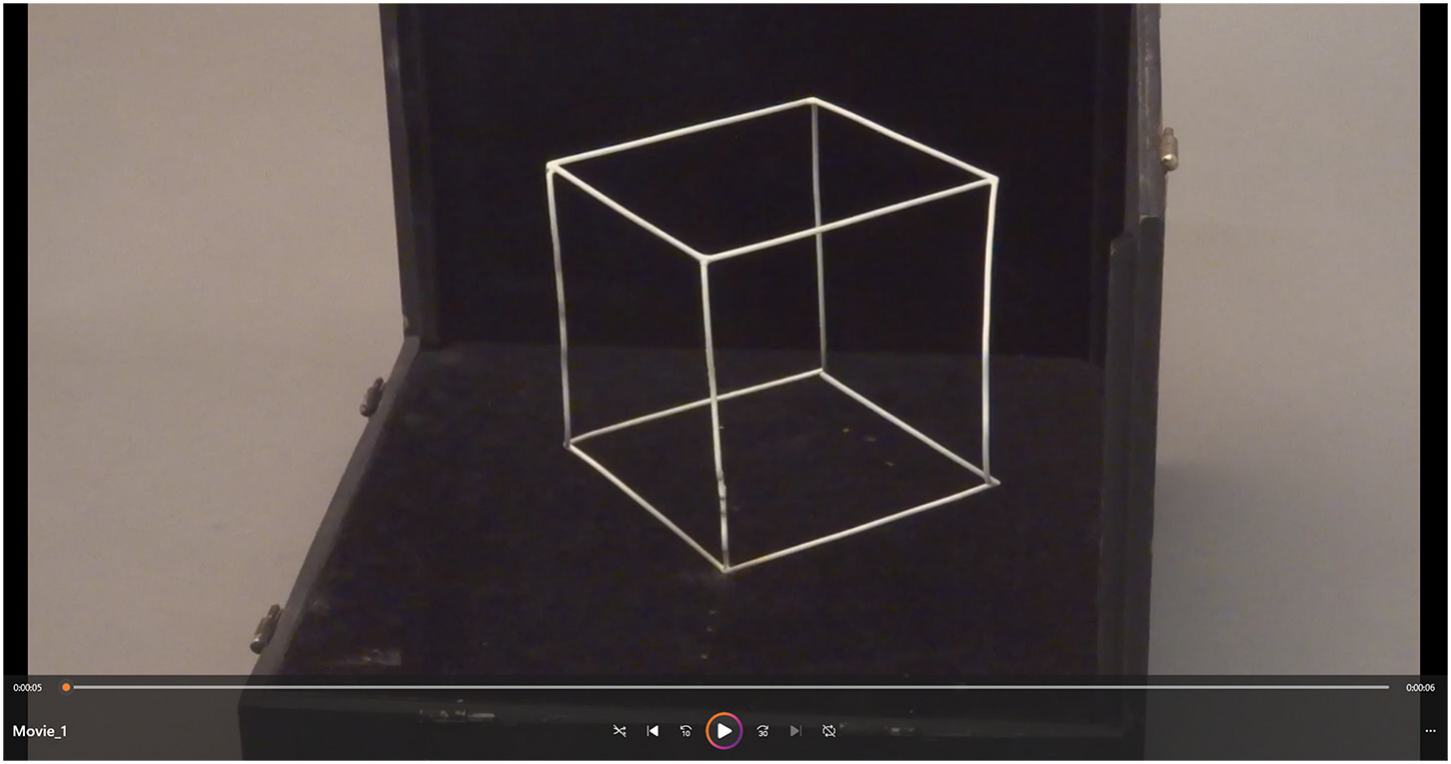
Presentation of the face-cube illusion in pushing the rod at the bottom of the box. A cube is perceived then a woman's face is seen.

This demonstration is close to other ambiguous patterns like the anamorphosis found in painting, a drawing presents a distorted image and appears in a specific form under a certain point of view (Topper, 2000). One of the most important aspects in the work done by Beuchet was to realize anamorphosis with 3D patterns. Beuchet (1996) indicated, from a personal communication, that he had accomplished preliminary works in using cube rotations in 1955. These previous patterns were not found, but some examples are available in a previous movie (available around 25 min) (Klementief, 1968).

It is well-known that the change of perspective was well developed in a previous demonstration by Aldebert Ames Jr. There are multiple well-known 3D phenomena, like the string-chair demonstration, in which a jumble of unconnected lines in 3D space look like a chair from one perspective; or the Ames window, in which a flat cardboard seems to be a rectangle when, in fact, it is a trapezoid (Ittelson, 1952). From a personal communication, Beuchet (1996) said he was unaware of the work of Aldebert Ames Jr. until 1955, when he was informed by Musatti and Kanisza of the fact that his demonstrations were similar to those developed by Aldebert Ames Jr. This meeting occurred during an international congress on cinema studies in Paris (Beuchet, 1996).

There is no doubt that Aldebert Ames Jr. influenced Jean Beuchet when looking at the similarities between the Ames room and Beuchet chair. Here, the face-cube illusion represents a more advanced version in comparison with previous works, because the face-cube illusion used two viewing points to perceive two different shapes. To our knowledge, there are no other 3D devices that allow one to obtain two visual objects from one pattern.

## Supplemental Material

**Figure:** Video 1.
